# Proton Magnetic Resonance Spectroscopy in Common Dementias—Current Status and Perspectives

**DOI:** 10.3389/fpsyt.2020.00769

**Published:** 2020-08-06

**Authors:** Stephan Maul, Ina Giegling, Dan Rujescu

**Affiliations:** University Clinic and Outpatient Clinic for Psychiatry, Psychotherapy and Psychosomatics, Martin Luther University Halle-Wittenberg, Halle, Germany

**Keywords:** proton magnetic resonance spectroscopy, Alzheimer’s disease, vascular dementia, dementia with Lewy bodies, frontotemporal dementia, biomarker

## Abstract

Dementia occurs mainly in the elderly and is associated with cognitive decline and impairment of activities of daily living. The most common forms of dementia are Alzheimer’s disease (AD), vascular dementia (VD), dementia with Lewy bodies (DLB), and frontotemporal dementia (FTD). To date, there are no causal options for therapy, but drug and non-drug treatments can positively modulate the course of the disease. Valid biomarkers are needed for the earliest possible and reliable diagnosis, but so far, such biomarkers have only been established for AD and require invasive and expensive procedures. In this context, proton magnetic resonance spectroscopy (^1^H-MRS) provides a non-invasive and widely available technique for investigating the biochemical milieu of brain tissue *in vivo*. Numerous studies have been conducted for AD, but for VD, DLB, and FTD the number of studies is limited. Nevertheless, MRS can detect measurable metabolic alterations in common dementias. However, most of the studies conducted are too heterogeneous to assess the potential use of MRS technology in clinical applications. In the future, technological advances may increase the value of MRS in dementia diagnosis and treatment. This review summarizes the results of MRS studies conducted in common dementias and discusses the reasons for the lack of transfer into clinical routine.

## Introduction

Dementia occurs mainly in the elderly and is associated with cognitive decline and impairment of activities of daily living. This leads to an increased need for care during the course of the disease. Dementia is therefore not only an enormous burden for the affected patients and their relatives, but also confronts social systems with great challenges, as the number of people suffering from dementia is expected to increase in the coming decades (1).

The most common cause of dementia is Alzheimer’s disease (AD), followed by vascular dementia (VD), dementia with Lewy bodies (DLB), and frontotemporal dementia (FTD) [for review see Cunningham et al. (2)]. Although these most common types of dementia differ in etiology, clinical symptoms, diagnostic findings, and treatment approaches, in many cases it is difficult to make a reliable diagnosis. A particular challenge is early detection and classification of cognitive impairments associated with discrete abnormalities that cannot be reliably distinguished clinically from age-related changes. The stage of slight symptoms is called mild cognitive impairment (MCI), MCI does not lead to limitations in activities of daily living, and it is a heterogeneous construct that has many underlying etiologies. In the context of dementia, MCI is seen as an intermediate predementia state of cognitive decline, but there are also stable and even reversible forms of MCI, which are not based on dementia neuropathology (3, 4). Additional tests using positron emission tomography (PET) and cerebrospinal fluid (CSF) are helpful for MCI and dementia diagnostics, but they are expensive, not widely available and invasive (5). However, there is still a lack of biomarkers which are easily available and allow early diagnosis and classification of dementia.

MR spectroscopy is a non-invasive *in vivo* method for measuring metabolite levels in various tissues based on the principles of nuclear magnetic resonance (NMR). More detailed literature on the physical background of MRS and on the clinical application in diseases of the central nervous system is provided by Ulmer and colleagues (6) and Öz et al. (7). Metabolites that are present in the brain at sufficiently high concentrations for quantification by MRS and have been commonly analyzed in dementia include N-acetylaspartate (NAA), myo-Inositol, total choline (tCho; primarily glycerophosphocholine and phosphocholine), and total creatine (tCr; creatine and phosphocreatine). Other metabolites less frequently investigated so far include glutamate+glutamine (Glx), *γ*-aminobutyric acid (GABA), and the antioxidant glutathione (GSH). NAA is highly concentrated in the brain and is mainly present in neurons, but also in oligodendrocytes. The exact function of NAA has not yet been clarified, and it has been hypothesized that it is involved in the energy metabolism of neuronal mitochondria, in the storage of acetyl coenzyme A, in signaling pathways and neurotransmission and in myelination processes (8–11). NAA is considered a marker of neuronal integrity, and reduced levels are found in various neuropathological conditions. However, it is unknown whether the reduced levels in MRS are due to a neuronal loss, neuronal dysfunction or disturbed NAA metabolism (8, 10). The tCho signal is mainly composed of glycerophosphocholine and phosphocholine, which are metabolites associated with the phospholipid metabolism of the cell membrane, whereby phosphocholine can be both a precursor or a degradation product and glycerophosphocholine is formed as a breakdown product (12). Disturbed tCho levels thus indicate an imbalanced cell membrane phospholipid metabolism, but MRS cannot determine whether this is driven by anabolic or catabolic pathways. In AD, elevated levels of glycerophosphocholine in the CSF were detected, which may indicate an increased membrane breakdown, possibly triggered by the activation of calcium-dependent phospholipase A_2_ (13, 14).

The sugar alcohol mI is considered a glial cell marker, as it occurs predominantly in glial cells and is involved in intracellular signaling pathways (15). The tCr peak is composed of creatine and phosphocreatine, which are involved in the energy metabolism, with the phosphorylated form of creatine serving as an energy buffer (16). In earlier studies, the tCr level has been described as relatively stable in dementia, AD and aging and was therefore often used as a reference for calculating metabolite ratios (e.g. NAA/tCr, mI/tCr) (17–19). This is problematic, as deviations in tCr levels were found in later studies [e.g. (20)] and tCr therefore does not appear to be a reliable reference marker. The Glx complex consists of signals of the metabolites glutamate (Glu), glutamine (Gln), and GABA and the individual peaks can only be separated at higher magnetic field strengths (3.0 T and higher) (21). Glu is the most important excitatory neurotransmitter in the brain which, after its release from the synaptic terminals, is transported into astrocytes, where it is converted into Gln and then it is made available to neurons again (Glu-Gln-cycle) (22). Although the functions of the different metabolites are not fully understood, which also makes the interpretation of metabolite abnormalities somewhat difficult, they can be useful as diagnostic markers and facilitate the understanding of disease-related biochemical alterations in brain tissue (8). For example, MRS can be used *in vivo* to determine which brain regions are affected by metabolic alterations particularly early in the course of the disease.

MRS acquisition distinguishes between single-voxel technique, in which signals from a previously selected voxel are obtained, and magnetic resonance spectroscopic imaging (MRSI), in which many voxels are acquired simultaneously (multi-voxel spectroscopy). Single-voxel spectroscopy (SVS) offers the advantage that scan times are shorter and the quantification of metabolites is more accurate, but only one brain region can be examined at a time. MRSI allows simultaneous acquisition of numerous brain regions with the disadvantage of being less precise and it demands a longer imaging time (23). In contrast to anatomical MR imaging, MRS requires suppression of the water signal to obtain measurable signals of the significantly lower concentrated metabolites. For this purpose, it is necessary to define sufficiently large voxels, which also increases scanning time (24). Other imaging parameters that differ between MRS studies include relaxation time (TR) and echo time (TE). A short TE leads to a high signal-to-noise ratio, so that more metabolite peaks can be acquired than with a longer TE. However, a short TE leads to an increased overlap of peaks, whereas some metabolites cannot be detected with a longer TE (e.g. mI and Glx). With higher TR values, better spectra can be acquired, but this is associated with longer imaging times (23, 25).

The use of brain MRS is no longer limited to research applications, but now complements clinical neuroimaging, for example in neuro-oncology and neuro-pediatrics (7). Numerous studies in dementia have been conducted in the past, but MRS has not yet found its way into clinical routine. There are numerous reasons for this and they will be discussed in this review. But first an overview of MRS studies in AD, DLB, VD, and FTD will be provided.

## Methods

For this review, a systematic literature research on PubMed was conducted using the keywords “proton magnetic resonance spectroscopy” in combination with one of the following terms: “Alzheimer’s disease”, “dementia with Lewy bodies”, “vascular dementia”, and “frontotemporal degeneration”. For VD (seven studies), DLB (nine studies), and FTD (seven studies) all studies published since 2000 were considered. For AD, significantly more studies have been published that were meta-analyzed in 2015 (26). The present review summarizes the findings of the meta-analysis and only lists MRS studies on AD published since 2015. MRS studies in MCI were only considered when a longitudinal design was used to verify conversion to dementia, which enabled early metabolite alterations to be detected. **Tables 1** **–** **8** provide a systematic overview of all identified MRS studies. As some studies report absolute metabolite levels and some provide metabolite ratios, two tables were compiled for each dementia considered. Significant differences between dementia and control groups are indicated by an arrow pointing up or down, whereas a horizontal arrow means that no differences were found. Results appear in several tables if more than one common dementia has been studied within the same study. Significant differences between different dementias are marked with footnotes in the tables. Studies that followed interventional approaches or investigated specific symptoms (e.g. depression) in dementia were not taken into account.

**Table 1:** Magnetic resonance spectroscopy studies in Alzheimer’s disease (studies reporting metabolite ratios).

Study	Parameters				Results					
	Field strength TR/TE (ms)technique	Sample	Area Voxel size (mm^3^)	Cohort	NAA/tCr	NAA/mI	mI/tCr	tCho/tCr	Glx/tCr	Others
Mitolo et al. (27)	1.5 T4,000/35 Single-voxel	Baseline: AD (n=25), MCI (n=38), HC (n=18)Clinical follow-up after 2 years:non-converter MCI (n=12), converter MCI (n=26),	Midline PCC (20x20x20)	AD	–	↓a	–	–	–	–
				Converter	–	↓a	–	–	–	–
				Non-converter	–	↔	–	–	–	–
Huang et al. (28)	3.0 T2,000/68 Singe-voxel	AD (n=17),MCI (n=21),HC (N=15)	Midline ACC (40x40x25)	AD	↓b	–	–	–	↓b	GABA+ ↔
				MCI	↔	–	–	–	↔	GABA+ ↔
			Right hippocampus (40x20x20)	AD	↔	–	–	–	↓	GABA+ ↔
				MCI	↔	–	–	–	↔	GABA+ ↔
Waragai et al. (29)	1.5 T2,000/25 Single-voxel	Baseline: HC (n=289)*	Midline PCC (20x20x20)	AD	↓	↓c	↑	–	–	–
				MCI	↓	↓c	↔	–	–	–
		7 years follow-up: AD (n=21), MCI (n=53), DLB (n=7), PD (n=8)		AD	↓	↓	↑d	–	–	–
				MCI	↔	↓	↑d	–	–	–
Fayed et al. (30)	3.0 T1,500/35 Single-voxel	aMCI (n=48) + clinical follow-up after 3 years: AD (n=15), aMCI (n=33)	Midline PCC (20x20x20)	AD converter	↔	–	mI ↔	–	↓	Glu/tCr ↓
			Left occipital cortex (20x20x20)		↔	–	mI ↓	–	↔	Glu/tCr ↔
Su et al. (31)	3.0 T3,450/35 Multi-voxel	AD (n=35),DLB (n=25),HC (n=34)	PCC	AD	↓	–	↓	↓	↓	–
			Thalamus		↓	–	↔	↓	↔	–
			Hippocampus		↔	–	↓	↔	↔	–
			Superior temporal cortex		↓	–	↓	↓	↔	–
			Prefrontal cortex		↓	–	↓	↓	↔	–
			Occipital cortex		↔e	–	↔	↔e	↔	–
			Caudate		↓	–	↓	↓	↔	–
			Corpus callosum		↓	–	↔	↓	↔	–
Guo et al. (32)	3.0 T1,500/35 Multi-voxel	Mild AD (n=15),aMCI (n=13),HC (n=16)	ACC (left+right) (10x10x15)	AD	–	–	-f	-f	–	–
			PCC (left+right) (10x10x15)		–	–	-f	–	–	–
Mandal et al. (33)	3.0 T2,500/120 Single-voxel	AD (n=21), MCI (n=22), HC (n=21)	Frontal cortex (left+right) (15,600)	AD	–	–	–	–	–	GSH ↓
				MCI	–	–	–	–	–	GSH ↓g
		AD (n=19), MCI (n=19), HC (n=28)	Hippocampus (left+right) (15,600)	AD	–	–	–	–	–	GSH ↓
				MCI	–	–	–	–	–	GSH ↓
Delli Pizzi et al. (17)	3.0 T2,000/39 Single-voxel	DLB (n=16), AD (n=16), HC (n=13)	Thalamus (left+right) (15x10x15)	AD	↔	–	–	↔h	–	tCr/H2O ↔
Bai et al. (34)	3.0 T2,000/68 Single-voxel	AD (n=15), HC (n=15)	Midline frontal lobe (30x30x30)	AD	–	–	–	–	–	GABA+/tCr ↔
			Midline parietal lobe (30x30x30)		–	–	–	–	–	GABA+/tCr ↓
Zhang et al. (35)	1.5 T2,000/30 Single-voxel	Baseline: MCI (n=57),Follow-up (after 18–32 months): DLB (n=10), AD (n=27), MCI (n=20)*	Midline frontal lobe (20x20x20)	AD	↔	–	↔	↔	–	–
			Midline PCC (20x20x20)		↔i	–	↔	↔	–	–
			Midline occipital lobe (20x20x20)		↔	–	↔	↔	–	–
Murray et al. (36)	3.0 T2,000/30 Single-voxel	AD (n=24), HC (n=17)	Midline PCC (20x20x20)	AD	↓	↓	↑	↔	–	–

ACC, anterior cingulate cortex; AD, Alzheimer’s disease; tCho, total choline; tCr, total creatine; DLB, dementia with Lewy bodies; GABA+, γ-aminobutyric acid, macromolecules and homocarnosine; Glu, glutamate; Glx, glutamate-glutamine; HC, healthy controls; MCI, mild cognitive impairment; mI, myo-Inositol; NAA, N-acetlyaspartate; PCC, posterior cingulate cortex; PD, Parkinson’s disease; TR, repetition time; TE, echo time.

↑ increased, ↓ decreased and ↔ unchanged metabolite ratios between AD and controls.

^*^Baseline differences were calculated on retrospective grouping according to clinical outcome.

^a^significantly lower ratios in AD and MCI-converter compared to stable MCI.

^b^significantly lower ratios in AD compared to MCI.

^c^significantly lower ratios in AD compared to progressor MCI at baseline.

^d^significantly higher ratios in AD compared to progressor MCI after 7 years.

^e^significantly lower ratios in AD compared to DLB.

^f^No comparisons of patients and controls were reported, but significant bilateral differences (left vs. right ACC, left vs. right PCC) were observed.

^g^significantly lower ratios in AD compared to MCI in the left frontal cortex.

^h^significantly lower ratios in AD compared to DLB in the right thalamus.

^i^significantly reduced ratios in MCI converted to AD compared to MCI converted to DLB.

**Table 2:** Magnetic resonance spectroscopy studies in Alzheimer’s disease (studies reporting absolute metabolite levels).

Study	Parameters				Results					
	Field strength TR/TE (ms) technique	Sample	AreaVoxel size (mm^3^)	Cohort	NAA	mI	tCho	tCr	Glx	Others
Joe et al. (37)	1.5 T1,500/30 Single-voxel	Preclinical ADAD (n=16),HC (n=11)	Left ACC (11x11x9)	ADAD	↓	↔	↔	↔	↓	–
			Right ACC (11x11x9)		↔	↔	↔	↔	↔	–
			Midline PCC (11x11x12)		↔	↔	↔	↔	↔	–
			Midline precuneus (11x11x12)		↓	↑	↑	↔	↔	–
Glodzik et al. (38)	3.0 T10,000/0 NA	AD (n=53), MCI (n=42), HC (n=102)	Whole brain	AD/MCI	↓	–	–	–	–	–
Zhong et al. (39)	3.0 T2,000/35 Single-voxel	DLB (n=19), AD (n=21), HC (n=18)	Midline PCC (20x20x20)	AD	↓	↔	↔	↔	↓	Glu ↓
			Midline occipital lobe (20x20x20)		↔a	↔	↔	↔a	↓	Glu ↓a

ACC, anterior cingulate cortex; AD, Alzheimer’s disease; ADAD, autosomal dominant AD; tCho, total choline; tCr, total creatine; DLB, dementia with Lewy bodies; Glu, glutamate; Glx, glutamate-glutamine; GSH, glutathione; HC, healthy controls; (a)MCI, (amnestic) mild cognitive impairment; mI, myo-Inositol; NAA, N-acetlyaspartate; PCC, posterior cingulate cortex; TR, repetition time; TE, echo time.

↑ increased, ↓ decreased and ↔ unchanged metabolite levels between AD and controls.

^a^ significantly higher levels in AD compared to DLB.

**Table 3:** Magnetic resonance spectroscopy studies in vascular dementia (studies reporting metabolite ratios).

Study	Parameters				Results					
	Field strength TR/TE (ms)technique	Sample	Area Voxel size (mm^3^)	Cohort	NAA/tCr	NAA/mI	mI/tCr	tCho/tCr	Glx/tCr	Others
Kantarci et al. (40)	NA2,000/30 Single-voxel	FTD (n=41),AD (n=121),DLB (n=20),VD (n=8),HC (n=206)	Midline PCC (20x20x20)	VD	↓	–	↔a	↔	–	–
Herminghaus et al. (41)	1.5 T1,500/20 Single-voxel	VD (n=15),AD (n=28), HC (n=15)	Midline frontal GM (8,000–12,600)	VD	↓b	–	↔	↑c	↔	–
			Midline parietal GM (8,000–12,600)		↓	–	↑	↑c	↑	–
			Left frontal WM (4,200–8,000)		↓	–	↑c	↑c	↔	–
			Left parietal WM (4,200–8,000)		↓	–	↑	↑c	↔	–
			Temporal gyrus (dominant hemisphere)		↓	–	↑	↔	↑	–
Waldman et al. (42)	1.0 T1,500/30 Single-voxel	VD (n=18), AD (n=20),Mixed dementia (n=6), HC (n=3)	Midline occipital lobe	VD	↔	–	↔b	↔	–	–

AD, Alzheimer’s disease; tCho, total choline; tCr, total creatine; DLB, dementia with Lewy bodies; FTD, Frontotemporal dementia; Glu, glutamate; Glx, glutamate-glutamine; GM, gray matter; HC, healthy controls; mI, myo-Inositol; NAA, N-acetlyaspartate; PCC, posterior cingulate cortex; TR, repetition time; TE, echo time; VD, vascular dementia; WM, white matter.

↑ increased, ↓ decreased and ↔ unchanged metabolite concentrations between VD and controls.

^a^significantly lower ratios in VD compared to AD and FTD.

^b^significantly lower ratios in VD compared to AD.

^c^significantly higher ratios in VD compared to AD.

**Table 4:** Magnetic resonance spectroscopy studies in vascular dementia (studies reporting absolute metabolite levels).

Study	Parameters				Results					
	Field strength TR/TE (ms)technique	Sample	Area Voxel size (mm^3^)	Cohort	NAA	mI	tCho	tCr	Glx	Others
Shiino et al. (43)	1.5 T2,000/30 Single-voxel	VD (n=30),AD (n=99),HC (n=45)	Left hippocampus (NA)	VD	↓a	↓b	↓	↓	↓	–
			Right hippocampus (NA)		↔a	↓b	↔	↓b	↓	–
			Midline PCC (NA)		↓	↓b	↔	↓	↔	–
Watanabe et al. (44)	1.5 T2,000/30 Single-voxel	VD (n=13),AD (n=30),HC (n=26)	Right hippocampus (3,000)	VD	↓a	↔b	↓	↔	–	–
			Left hippocampus (3,000)		↓a	↔	↔	↔	–	–
			Midline PCC (5,000)		↔	↔b	↔	↔	–	–
			Midline occipital lobe (6,000)		↓	↔b	↔	↔	–	–
			Right anterior periventricular WM (4,000)		↓b	↓b	↓b	↓b	–	–
			Left anterior periventricular WM (4,000)		↓b	↓b	↓b	↓b	–	–
			Right posterior periventricular WM (4,000)		↓	↓b	↓b	↓	–	–
			Left posterior periventricular WM (4,000)		↓b	↓b	↓b	↓b	–	–
Schuff et al. (20)	1.5 T2,000/30 Multi-voxel	VD (n=13),AD (n=43),HC (n=52)	Frontal lobe (left+right) (7.8x7.8x15)	VD	↓b	–	↔	↔	–	–
			Parietal lobe (left+right) (7.8x7.8x15)		↓b	–	↔	↔	–	–
			Temporal lobe (left+right) (8.5x8.5x15)		↔	–	↔	↔	–	–
Capizzano et al. (45)	1.5 T1,800/135 Single-voxel+Multi-voxel	AD (n=18), Dementia with lacunes (n=11), MCI with lacunes (n=14), HC (n=20)	Frontal lobe (8x8x15)	Dementia + lacunes	↓	–	↔	↔	–	–
			Parietal lobe (8x8x15)		↓	–	↔	↔	–	–
			Hippocampus (8.75x8.75x15)		↔	–	↔	↔	–	–
			WM		↓	–	↔	↔	–	–

AD, Alzheimer’s disease; tCho, total choline; tCr, total creatine; Glx, glutamate-glutamine; GM, gray matter; HC, healthy controls; MCI, mild cognitive impairment; mI, myo-Inositol; NA, Not applicable; NAA, N-acetlyaspartate; PCC, posterior cingulate cortex; TR, repetition time; TE, echo time; VD, vascular dementia; WM, white matter.

↑ increased, ↓ decreased and ↔ unchanged metabolite levels between VD and controls.

^a^ significantly higher levels in VD compared to AD.

^b^ significantly lower levels in VD compared to AD.

**Table 5:** Magnetic resonance spectroscopy studies in dementia with Lewy bodies (studies reporting metabolite ratios).

Study	Parameters				Results					
	Field strength TR/TE (ms)technique	Sample	Area Voxel size (mm^3^)	Cohort	NAA/tCr	NAA/mI	mI/tCr	tCho/tCr	Glx/tCr	Others
Waragai et al. (29)	1.5 T2,000/25 Single-voxel	Baseline: HC (n=289)*	Midline PCC (20x20x20)	DLB	↔	↓a	↔	–	–	–
				PD	↔	↔a	↔	–	–	–
		7 years follow-up: AD (n=21), MCI (n=53), DLB (n=7), PD (n=8)		DLB	↓b	↓	↑	–	–	–
				PD	↔b	↔	↔	–	–	–
Su et al. (31)	3.0 T3,450/35 Multi-voxel	AD (n=35),DLB (n=25),HC (n=34)	PCC	DLB	↓	–	↓	↔	↓	–
			Thalamus		↓	–	↔	↓	↔	–
			Hippocampus		↔	–	↓	↔	↓	–
			Superior temporal cortex		↓c	–	↓	↓	↓	–
			Prefrontal cortex		↓	–	↔	↓	↔	–
			Occipital cortex		↔c	–	↑	↔c	↔	–
			Caudate		↓	–	↓	↓	↓	–
			Corpus callosum		↓	–	↔	↔	↔	–
Delli Pizzi et al. (17)	3.0 T2,000/39 Single-voxel	DLB (n=16), AD (n=16), HC (n=13)	Thalamus (left+right) (15x10x15)	DLB	↓	–	–	↑d	–	tCr/H2O ↔
Xuan et al. (46)	1.5 T1,500/135 Single-voxel	DLB (n=8),HC (n=8)	Hippocampus (left+right)	DLB	↓	–	–	↔	–	–
Zhang et al. (35)	1.5 T2,000/30 Single-voxel	Baseline: MCI (n=57),Follow-up (after 18–32 months): DLB (n=10), AD (n=27), MCI (n=20)*	Midline frontal lobe (20x20x20)	DLB	↔	–	↔	↔	–	–
			Midline PCC (20x20x20)		↔e	–	↔	↔	–	–
			Midline occipital lobe (20x20x20)		↔	–	↔	↔	–	–
Graff-Radford et al. (47)	1.5 T2,000/30 Single-voxel	DLB (n=34), AD (n=35), HC (n=148)	Midline frontal lobe (20x20x20)	DLB	↔	–	↔	↔	–	–
			Midline PCC (20x20x20)		↔	–	↑	↑	–	–
			Midline occipital lobe (20x20x20)		↓	–	↔	↔	–	–
Kantarci et al. (40)	NA2,000/30 Single-voxel	FTD (n=41),AD (n=121),DLB (n=20),VD (n=8),HC (n=206)	Midline PCC (20x20x20)	DLB	↔f	–	↔g	↑	–	–
Molina et al. (48)	1.5 T1,700/35 Single-voxel	DLB (n=12), HC (n=11)	Left centrum semiovale (WM) (4,840)	DLB	↓	–	↔	↓	↓	–
			Midline parietal region (GM) (5,830)		↔	–	↔	↔	↔	–

AD, Alzheimer’s disease; tCho, total choline; tCr, total creatine; DLB, dementia with Lewy bodies; FTD, Frontotemporal dementia; Glx, glutamate-glutamine; GM, gray matter; HC, healthy controls; MCI, mild cognitive impairment; mI, myo-Inositol; NA, Not applicable; NAA, N-acetlyaspartate; PCC, posterior cingulate cortex; PD, Parkinson’s disease; TR, repetition time; TE, echo time; VD, vascular dementia; WM, white matter.

↑increased, ↓ decreased and ↔ unchanged metabolite concentrations between DLB and controls.

^*^Baseline differences were calculated on retrospective grouping according to clinical outcome.

^a^significantly lower ratios in DLB compared to PD at baseline.

^b^significantly lower ratios in DLB compared to PD after 7 years.

^c^significantly higher ratios in DLB compared to AD ^d^ in the right thalamus.

^e^significantly lower ratios in MCI converted to AD compared to MCI converted to DLB.

^f^significantly lower ratios in AD and FTD compared to DLB.

^g^significantly lower ratios in DLB compared to FTD.

**Table 6:** Magnetic resonance spectroscopy studies in dementia with Lewy bodies (studies reporting absolute metabolite levels).

Study	Parameters				Results					
	Field strength TR/TE (ms)technique	Sample	Area Voxel size (mm^3^)	Cohort	NAA	mI	tCho	tCr	Glx	Others
Zhong et al. (39)	3.0 T2,000/35 Single-voxel	DLB(n=19), AD (n=21), HC (n=18)	PCC (20x20x20)	DLB	↓	↔	↔	↔	↓	Glu ↓
			Bilateral occipital lobe (20x20x20)		↓a	↔	↔	↓a	↓	Glu ↓a

AD, Alzheimer’s disease; tCho, total choline; tCr, total creatine; DLB, dementia with Lewy bodies; Glu, glutamate; Glx, glutamate-glutamine; HC, healthy controls; mI, myo-Inositol; NAA, N-acetlyaspartate; PCC, posterior cingulate cortex; TR, repetition time; TE, echo time.

↑ increased, ↓ decreased and ↔ unchanged metabolite concentrations between AD and controls.

^a^significantly lower levels in DLB compared to AD.

**Table 7:** Magnetic resonance spectroscopy studies in frontotemporal dementias (studies reporting metabolite ratios).

Study	Parameters				Results			
	Field strength TR/TE (ms) technique	Sample	Area Voxel size (mm^3^)	Cohort	NAA/tCr	NAA/mI	mI/tCr	tCho/tCr
Chen et al. (49)	3.0 T2,000/30 Single-voxel	MAPT mutation carriers (n=19), HC (N=25)	Midline superior frontal gyrus (20x20x20)	Symptomatic (n=10)	↓	↓	↑	–
				Asymptomatic(n=9)	↓	↓	↔	–
Kantarci et al. (50)	3.0 T2,000/30 Single-voxel	MAPT mutation carriers (n=24), HC (N=24)	Midline PCC (20x20x20)	Symptomatic (n=10)	↓a	↓a	↑	–
				Asymptomatic(n=14)	↔a	↓a	↑	–
Coulthard et al. (51)	1.5 T2,000/30 Single-voxel	FTD (n=7),HC (n=11)	Midline frontal lobe (40x40x20)	FTD	↓	–	↑	–
			Left parietal lobe (40x30x20)		↔	–	↔	–
			Right temporal lobe (40x20x20)		↓	–	↔	–
Mihara et al. (52)	3.0 T6,000/25 Multi-voxel	FTD (n=6),PSP (n=3),CBD (n=1),AD (n=8),HC (n=14)	Midline ACC (20x20x20)	FTD	↓	–	↔	↔
			Midline PCC (20x20x20)		↓	–	↑	↔
			Perieto-occipital WM (left) (20x20x20)		↔	–	↔	↔
			Frontal WM (left) (20x20x20)		↓b	–	↑b	↑
Kantarci et al. (40)	NA2,000/30 Single-voxel	FTD (n=41),AD (n=121),DLB (n=20),VD (n=8),HC (n=206)	Midline PCC (20x20x20)	FTD	↓c	–	↑d	↑
Kizu et al. (53)	1.5 T2,000/135 Multi-voxel	FTD (n=6), AD (n=6),HC (n=5)	PCC (left+right) (18x18x10)	FTD	↓	–	–	↔

ACC, anterior cingulate cortex; AD, Alzheimer’s disease; CBD, corticobasal degeneration; tCho, total choline; tCr, total creatine; DLB, dementia with Lewy bodies; FTD, Frontotemporal dementia; HC, healthy controls; mI, myo-Inositol; NA, Not applicable; NAA, N-acetlyaspartate; PCC, posterior cingulate cortex; PSP, progressive supranuclear palsy; TR, repetition time; TE, echo time; VD, vascular dementia; WM, white matter.

↑ increased, ↓ decreased and ↔ unchanged metabolite ratios between FTD and controls.

^a^significantly lower ratios in symptomatic compared to presymptomatic mutation carriers.

^b^significant differences between FTD and AD.

^c^significantly lower ratios in FTD compared to DLB.

^d^significantly higher ratios in FTD compared to DLB and VD.

**Table 8:** Magnetic resonance spectroscopy studies in frontotemporal dementias (studies reporting absolute metabolite levels).

Study	Parameters				Results			
	Field strength TR/TE (ms)Technique	Sample	Area Voxel size (mm^3^)	Cohort	NAA	mI	tCho	tCr
Chawla et al. (54)	3.0 T1,700/30 Multi-voxel	FTD (n=26), HC (n=10)	Dorsolateral PFC (left+right) (10x9.4x20)	FTD	↓	↑	↑	↓
			Motor cortex (left+right) (10x9.4x20)		↓	↔	↑	↔
			Parietal cortex (10x9.4x20)		↔	↔	↔	↔

tCho, total choline; tCr, total creatine; FTD, Frontotemporal dementia; HC, healthy controls; mI, myo-Inositol; NAA, N-acetlyaspartate; PFC, prefrontal cortex; TR, repetition time; TE, echo time.

↑ increased, ↓ decreased and ↔ unchanged metabolite levels between FTD and controls.

### Alzheimer’s Disease

AD is by far the most common form of dementia in the elderly and leads to progressive cognitive decline, often initially affecting memory function. Typical histological findings are extracellular amyloid plaques and intracellular neurofibrillary tangles (55). In recent years, it has become increasingly apparent that early detection of AD seems to be an important prerequisite for pharmacological treatment (56). Thus, current clinical trials are focusing on individuals with an increased risk of AD, who do not yet have symptoms (preclinical AD) or who are at the stage of MCI (57). The use of biomarkers increases diagnostic accuracy, which is why the framework on diagnostic criteria for AD published by the National Institute on Ageing–Alzheimer’s Association (NIA-AA) in 2018 recommends the use of biomarkers for AD diagnosis, particularly in the context of research projects (58). However, the best validated biomarkers to date can be determined either invasively by lumbar puncture (amyloid-β, tau, phospho-tau) or by using expensive imaging techniques that are not available on a large scale (amyloid PET). The huge dilemma is that there are still no easily and widely available biomarkers that would allow early and reliable diagnosis.

In 2015, a meta-analysis of 38 MRS studies in AD was published, with most MRS studies using 1.5 T MRI (26). Although more studies on this topic have been identified, not all of them could be included in the analyses due to the heterogeneity of the studies (e.g. lack of information on acquisition parameters or missing data needed to calculate effect sizes). Meta-analysis data are available for the four most frequently investigated brain regions, including the posterior cingulate cortex (PCC) (investigated in 17 studies), hippocampus (nine studies), and temporal and parietal lobes (seven studies each). Consistently, the NAA/tCr ratio or NAA level were significantly lowered in the four examined brain regions (Hedges g between −1.29 and −0.83). For the other metabolites analyzed in the meta-analysis, the results were more heterogeneous. Although a significantly increased mI/tCr ratio was found in the PCC (g=0.83), the effect was considerably lower when using absolute mI levels (g=0.32). A moderate increase in the PCC was found for tCho/tCr (g=0.48), while no significant difference was found for absolute tCho levels. In addition, an increased mI level in parietal gray matter was obtained in AD patients, whereas data for the other regions and metabolites were not sufficient for the meta-analysis. In summary, only for NAA a significant decrease in the investigated brain regions has been determined, while for the other metabolites data are less clear (e.g. due to a lack of studies in which these metabolites were acquired and discrepancies between reported metabolite ratios and absolute levels).

Since the publication of the meta-analysis, 14 MRS studies on AD have been published, including four studies with a longitudinal study design (see **Tables 1** and **2**). Consistent with the results of the meta-analysis, altered NAA and mI metabolites in the PCC were observed in four studies (27, 29, 36, 39). However, discrepant results were found in the study conducted by Su et al. (31), which revealed decreased NAA/tCr ratios in several brain areas and, also decreased mI/tCr and tCho/tCr ratios in the PCC, hippocampus, temporal, and frontal cortex. This opposite effect to other studies also occurred in DLB patients investigated in this study and is therefore discussed in the DLB section. Some studies also examined brain areas that were previously less in the focus. The occipital lobe was investigated in three recent studies, with no differences found between AD and controls (31, 35, 39). In the frontal cortex, Zhang et al. (35) detected no metabolite differences. This is consistent with a previous report (59), while the aforementioned study by Su et al. (31) obtained decreased NAA/tCr, mI/tCr, and tCho/tCr ratios.

Further studies focused on metabolites whose levels can be determined at higher field strengths (3.0 T or more) by better spectral resolution. Thus, Bai et al. (34) found a reduced level of γ-aminobutyric acid (GABA+), the major inhibitory neurotransmitter in the brain, in the parietal lobe of AD patients, while no differences between AD patients and healthy controls were found in the frontal lobe, hippocampus and ACC (28, 34). In addition, Mandal et al. (33) detected reduced levels of glutathione (GSH), an important antioxidant present in high concentrations in the brain (60), within the hippocampus and frontal cortex of AD patients. Chiang et al. (61) also investigated GSH levels, but in cognitively unimpaired older volunteers (average age: 63 ± 5 years), and found a negative correlation of amyloid load measured by Pittsburgh Compound B PET imaging with GSH levels in the temporal (r=−0.51) and parietal lobe (r=−0.47). The implementation of modern MRS techniques thus enables the detection of further metabolites in the brain tissue. However, its potential benefit must be verified in further studies.

It is also worth taking a look at recent studies in which the clinical outcome was determined in a prospective study design, with three studies involving patients with MCI (27, 30, 35) and one study involving cognitively healthy elderly individuals (29). The PCC was investigated in all studies. Waragai and colleagues and Mitolo and colleagues found significant metabolite deviations in subjects who progressed to AD, but Zhang et al. and Fayed and colleagues observed no significant differences (except for a lowered Glu level in the latter study). However, in asymptomatic carriers of mutations associated with autosomal dominant AD (ADAD), evidence of early metabolic alterations was found, with decreased NAA and increased mI and tCho levels in the precuneus reported (37).

In summary, there is consistent data on AD associated metabolic deviations that occur predominantly in brain areas typically affected in AD (hippocampus, temporal, and parietal lobe) with evidence of early detectable abnormalities. Replications in larger samples and the investigation of metabolites, which are facilitated by improved MRI technology, are necessary.

### Vascular Dementia

VD is the second most common form of dementia after AD and in addition to rare causes, subcortical ischemia (small vessel dementia) and cerebral infarcts (multi-infarct dementia) play the most important role in VD pathology. For both VD and AD, the presence of vascular risk factors is of relevance. There are patients for whom a clear classification is not possible or who suffer from mixed dementia. However, the classification is important for the treatment of patients, as the therapy regimes differ in both diseases [for review see O’Brien and Thomas (62)].

A total of seven MRS studies were identified in which patients with VD were recruited (see **Tables 3** and **4**). Among these studies, frontal and parietal lobes, hippocampus (four studies each) and PCC (three studies) were the most frequently examined. In all studies that examined these four brain regions, a reduction in the NAA/tCr ratio or NAA level was found (20, 40, 41, 43–45). For mI/tCr (or absolute mI level) and tCho/tCr ratios (or absolute tCho level), the results were inconsistent, with one study reporting increased levels in the frontal and parietal lobe (41), two studies reporting decreased levels in the hippocampus and in frontal and parietal white matter respectively (43, 44), and two studies reporting no differences in the frontal and parietal lobe (20, 45). Schiino et al. (43) discussed this fact, but failed to identify relevant differences in study design that could explain these different results. Interestingly, both Schiino et al. (43) and Watanabe et al. (44) report metabolite ratios as well as absolute metabolite levels, whereby the significant differences between VD patients and healthy controls almost completely disappear when using the ratio values. Since both studies obtained lowered tCr levels, this may indicate that tCr is not suitable as an internal reference (at least for VD). This could explain the discrepancies with the study by Herminghaus et al. (41), in which only ratios were reported. In addition, the white matter hyperintensity load within the voxels may have an influence, but in an early study no deviations of the metabolites tCho and mI between white matter hyperintensities and normal appearing white matter were found (20). In all MRS studies on VD, patients with AD were also included, with six of the seven studies showing significant differences between both disorders. Metabolic alterations (NAA, mI, tCho) from the control group were more pronounced in VD in the frontal and parietal lobes (in particular in white matter) and in AD in the hippocampus and PCC (20, 40–44).

In recent years, studies have been conducted in patients with vascular MCI (vMCI), the equivalent of aMCI in AD, which is considered the early form of cognitive impairment due to cerebrovascular disease. Reduced NAA/tCr ratios were reported in three studies, affecting the frontal white matter, PCC, and thalamus. Largely no differences were observed for other metabolites compared to the healthy controls (63–65), indicating early detectable deviations. One limitation is that in the study by Liu et al. (65) significant differences between vMCI and controls were only found when considering metabolite ratios, but not absolute metabolite levels. The other two studies did not report absolute metabolite levels, therefore further studies are needed.

### Dementia With Lewy Bodies

DLB is a neurodegenerative disorder and, like Parkinson’s disease (PD) and multiple system atrophy, belongs to α-synucleinopathies. The common feature of this heterogeneous group of disorders is the detection of Lewy bodies in various brain regions and the occurrence of parkinsonism, cognitive decline, and visual hallucinations [for review see Hansen et al. (66)]. However, AD-typical amyloid β plaques and tau pathology are also observed in many DLB patients (67–69). In contrast to AD, antipsychotic treatment with neuroleptics is contraindicated due to inducing extrapyramidal symptoms, which emphasizes the importance of early detection of DLB.

A total of nine MRS studies in which DLB patients were examined have been identified. Seven studies also examined patients with AD and one study included AD, FTD, and VD patients (see **Tables 5** and **6**). Overall, the results for DLB are inconsistent, which is evident when looking at the PCC. The PCC was investigated in six studies: Three studies obtained reduced NAA/tCr ratios and three studies found no difference between patients and healthy subjects (31, 35, 39, 40, 47, 54). Metabolic alterations in the occipital lobe were observed in three studies (31, 39, 47), while one study found no differences (35). This study included MCI patients and deviations may not yet be detectable due to the early stage of the disease. Other brain regions affected by metabolite alterations were the thalamus (17, 31) and the hippocampus (31, 46), whereas no differences were found for the frontal lobe in two studies (35, 47). An exception is the study by Su et al. (31), in which, in contrast to the other studies, lowered tCho/tCr and mI/tCr ratios were obtained in several brain regions (PCC, thalamus, hippocampus, temporal cortex, prefrontal cortex, basal ganglia). The authors discussed these contradictory results with possibly decreasing glial cell activation and inflammation (and thus decreasing metabolite levels) within these brain regions over the course of the disease. According to cognitive severity measured with the Mini Mental Status Examination (MMSE), however, DLB patients were more affected in other studies (Su et al.: 20.3/30 points; Delli Pizzi et al.: 17.9/30, Xuan et al.: 17.0/30 points), therefore this assumption is purely speculative and further studies are necessary. Technical reasons are also conceivable, as the study by Su et al. was the only one using multi-voxel technology.

Significant differences between DLB and AD were detected in the occipital lobe with significantly lower NAA, tCr, and Glu levels in DLB patients in a study by Zhong et al. (39). Significant deviations of NAA/tCr and mI/tCr from AD in the occipital lobe were also found in another study (47), which additionally considered post-mortem autopsy confirmation. The authors explained this approach with the fact that DLB is often accompanied by AD-typical pathology. Interestingly, those DLB patients without histological evidence of AD pathology showed preserved occipital NAA/tCr ratios compared to healthy controls, while patients with mixed DLB/AD tended to have reduced NAA/tCr levels. Nevertheless, it remains questionable whether it is possible to distinguish between DLB and AD by using MRS, as the results are not conclusive for the two most frequently examined brain regions (PCC and occipital lobe) (17, 31, 39, 40).

Two studies used a longitudinal design. Waragai et al. (29) included 289 cognitive healthy controls and performed MRS at baseline and 7 years later, and Zhang et al. (35) performed MRS in 57 MCI patients at baseline and assessed clinical outcome 18–32 months later. In the first study, no differences in metabolites between DLB and AD, but between DLB and PD (at baseline lower NAA/mI ratio in DLB and after 7 years lower NAA/tCr ratio in DLB in the PCC), have been detected. In the second study, however, it was possible to distinguish MCI patients who converted to AD from those who converted to DLB (lower NAA/tCr ratio in the PCC of AD converters). Significant differences at baseline between individuals who have progressed to DLB and individuals who remained cognitively unaffected were only found in the study by Waragai et al. (reduced NAA/mI ratio in PCC). Therefore, it is doubtful whether early detection of DLB using MRS is possible. A limiting factor is that only a small proportion of the individuals examined have converted to dementia. Although the longitudinal approach is promising, much larger samples must be recruited.

### Frontotemporal Dementia

Frontotemporal dementia (FTD) is a neurodegenerative disorder that is classified into different variants according to its predominant clinical symptoms: the behavioral subtype, primary progressive aphasia (semantic and nonfluent agrammatic variant) and FTD with motor neuron disease. Other diseases related to FTD are corticobasal degeneration (CBD) and progressive supranuclear palsy (PSP) [for review see Finger (2016) (70)]. Typically, FTD occurs earlier than other common dementias with the highest incidence rate between 45 and 64 years (71).

Although FTD is the second most common early-onset neurodegenerative dementia, only seven, mostly small sample size MRS studies have been identified in the literature search (see **Tables 7** and **8**).

The most consistent results were found in the frontal lobe and PCC, which were investigated in four studies each, and all showed decreased NAA/tCr (or NAA levels) and increased mI/tCr ratios (or mI levels) (40, 49–52, 54, 71). The same pattern of metabolic differences was found in the temporal lobe, motor cortex, and ACC, but these brain regions were only investigated in one study each (51, 52, 54). Three studies investigated the parietal lobe, where no metabolic differences in FTD patients were observed compared to healthy controls (51, 52, 54). Thus, metabolic alterations seem to occur particular in brain regions predominantly affected by FTD (frontal and temporal lobes).

More frequently than other common dementias, FTD shows a dominant inheritance pattern (10–23 % of the patients), with mutations in the *MAPT* gene (microtubule associated protein tau) being found in several affected families [for review see Gossye et al. (72)]. In two studies, asymptomatic and symptomatic *MAPT* mutation carriers were investigated, providing insights into early metabolic changes in the brains of affected individuals. Significant differences in metabolites for both symptomatic and asymptomatic mutation carriers compared to non-carriers were found in the frontal lobe (49) and the PCC (50), indicating early detectable alterations. In addition, three studies compared FTD with other dementias. While significant differences between FTD, DLB, and VD, but not between FTD and AD (40, 53), were shown in the PCC, another study found differences between FTD and AD in the frontal white matter (WM) (52). However, current data are too limited and samples from previous studies are too small to draw conclusions on the value of MRS in distinguishing FTD from other dementias or to detect it at an early stage.

## Discussion

Dementias are caused by neurodegenerative processes in the brain and lead to progressive cognitive decline. By far the most common form is AD, followed by VD, DLB, and FTD. Even if no causal treatments are available for these diseases, a reliable diagnosis as early as possible plays a major role in modulating the course of the disease through drug and non-drug therapies and enable patients to create a self-determined future. The establishment of validated biomarkers for AD and non-AD neurodegenerative disorders remains a major challenge (73).

MRS provides a non-invasive, widely available and cost-effective technique to investigate neurometabolites in brain tissue *in vivo*. However, the use of MRS technology in the field of neurodegenerative disorders has been limited primarily to research activities. Among MRS studies in dementia, by far the most were conducted in AD, the results of which were analyzed in a meta-analysis published in 2015 (26). The most robust results with a reduced NAA/tCr and increased mI/tCr and tCho/tCr ratios were obtained in the PCC. The results for the other brain areas analyzed (hippocampus, temporal and parietal lobes) were less clear and, only a consistent reduction in NAA/tCr ratios has been demonstrated.

For this review, 14 studies on AD were identified which have been published since the meta-analysis and largely confirmed the previous results (see **Tables 1** and **2**). Thus, metabolic alterations in AD seem to occur predominantly in those brain regions that are particularly affected by the disease. This is supported by some recent studies showing the frontal and occipital lobes apparently less affected (35, 39). There is still controversy over the significance of reduced NAA levels. For example, a decreasing number of neurons in the affected tissues or a lowered neuronal energy status are being discussed (8).

There are considerably fewer MRS studies on other common dementias (in the PubMed search seven studies in VD, nine studies in DLB, and seven studies in FTD were identified, which have been published since 2000). Compared to AD, metabolic changes in VD do not seem to be restricted to certain brain regions (**Tables 3** and **4**), and many studies focus on the white matter, which appears to be particularly affected in VD (74). In DLB, metabolite deviations were reported in the occipital lobe, thalamus, and hippocampus (see **Tables 5** and **6**), but metabolic alterations were also found in the PCC. Besides metabolic alterations in the frontal and temporal lobes, the PCC was also affected in FTD (see **Tables 7** and **8**). Although significant metabolite differences between dementias were found in comparative MRS studies [e.g. metabolic alterations in the PCC were more pronounced in AD than in DLB (35, 40)], metabolites acquired in MRS are not dementia-specific and per se do not allow any conclusion on the underlying pathology. Topographic patterns of metabolite alterations across the brain may be helpful in distinguishing dementias, but this needs to be investigated systematically (e.g. by using MRSI technology), as some brain regions were underrepresented in previous MRS studies.

Besides the use of MRS to differentiate dementia, another interesting aspect is the detection of metabolic alterations in early disease stages. This is a particular challenge, as mild cognitive complaints caused by incipient dementia are difficult to distinguish from non-pathological ageing processes (4). Previous studies indicate that metabolic changes can be detected in preclinical (29, 37, 49) and MCI stages (27, 38). However, in order to assess the potential use of MRS for early detection, large-scale, preferably longitudinal studies are necessary.

Despite some consistent findings obtained in previous MRS studies in common dementias, there are numerous limitations restricting the validity of the results and hampering the transfer of MRS technology to clinical application. This will be addressed in the following. The individual studies differ, in part significantly, in terms of their acquisition parameters, which makes a direct comparison difficult. In most studies, single-voxel spectroscopy (SVS) was used, in which only signals from one selected brain region are obtained. Although this enables a more accurate quantification of metabolites, only a limited number of brain regions can be examined in order to keep the scan time reasonable for patients. As a consequence, the focus was on many different brain regions, and replications were lacking. Compared to SVS, magnetic resonance spectroscopic imaging (MRSI) has an advantage, because it allows the acquisition of numerous voxels simultaneously (23). In addition, the voxel size must have a certain volume to achieve a good quality signal to noise ratio. This, however, impairs the acquisition of signals from small anatomical structures such as the hippocampus, especially if it is atrophied (75). Partial volume effects are expected in many MRS studies in dementia, as often voxels were used for MRS acquisition, which included surrounding tissue and cerebral fluid in addition to target structures. The increased use of devices with higher magnetic field strengths (3 T and higher) can partially counteract this problem by enabling smaller voxel sizes. Another advantage is that 3 T scanners provide a better spectral resolution, which allows the quantification of further metabolites in the brain (76).

The problem has already been mentioned that the metabolites detectable with MRS are not disease specific and that alterations have been observed in all dementias. Furthermore, many studies report on metabolite ratios rather than absolute metabolite levels. As an internal reference, total Cr (consisting of phosphocreatine and creatine) is usually used, since it has been shown to be relatively stable in AD (17, 18) and with age (19). However, there are also studies that found fluctuations in tCr levels in dementia (44, 48, 77). In the case of fluctuating tCr levels, relative ratios may lead to artificially altered group differences, as in the study of Watanabe and colleagues (44), where a dissociation between absolute metabolite levels and ratios has occurred. As an alternative to tCr, the unsuppressed tissue water signal can be used as an internal reference as it is subject to few pathology associated fluctuations (78). Also the authors of the meta-analysis in AD recommend the preferred use of absolute metabolite levels (26).

A general problem of almost all MRS studies in dementia is the small sample size and the classification of patients, which is mostly based on clinical criteria. Only five studies used additional tests to increase diagnostic certainty: in the study by Waragai et al. (29), CSF biomarkers (Aβ40, Aβ42, and phospho-Tau) and ^123^I-MIBG myocardial scintigraphy were used to confirm the clinical diagnosis in those participants who had progressed to MCI or dementia during the 7-year follow-up period. Murray and colleagues (36) performed post-mortem histopathological examinations to verify the clinical diagnosis of AD. And the presence of genetic origins was demonstrated in three further studies by the detection of disease-causing mutations [autosomal-dominant AD (37) and *MAPT* mutations (50, 63)]. The vast majority of studies did not use complementary biomarkers and the diagnosis relied exclusively on clinical criteria, which may have contributed to the heterogeneity of the study results. In order to make a reliable diagnosis, extensive and invasive diagnostic tests or post-mortem confirmation by autopsy are necessary, which would probably go beyond the scope of what is feasible for many research projects. This is a dilemma, because reliable diagnosis without valid biomarkers will continue to be a difficulty in these studies. Furthermore, effect sizes are often not reported and some studies do not provide group mean values, which also limits the ability to assess and compare between studies. And finally, correction for multiple statistical tests performed within a study is usually omitted, but this entails the risk of alpha error accumulation.

These numerous limitations make ^1^H-MRS currently not suitable for diagnostics and classification of dementia in clinical routine. New technologies, such as MEGA-PRESS spectroscopy, which facilitate the quantification of metabolites (e.g. GABA and GSH) that cannot be differentiated with conventional methods (79, 80), high speed magnetic resonance spectroscopic imaging (25), and 3D magnetic resonance spectroscopic imaging (81) may help to overcome the obstacles mentioned above. This requires large-scale, multi-center studies, which are conducted under standardized conditions (7). Longitudinal studies with a sufficiently long observation period are also necessary to assess early metabolite alterations and changes over time in patients with dementia.

## Conclusions

Proton magnetic resonance spectroscopy (^1^H-MRS) is a cost-effective, non-invasive and widely available technique for *in vivo* measurements of the biochemical milieu in brain tissue. MRS studies on dementia have been conducted in particular for AD, whereas studies on VD, DLB, and FTD are relatively rare. Alterations of several metabolic markers have been identified in all common dementias, which are already detectable in preclinical and early stages of the disease. The most consistent findings have been obtained for AD, where a decrease in NAA and an increase in mI and tCho levels in the posterior cingulate cortex were demonstrated in numerous studies and confirmed in a meta-analysis. Further brain regions and the other dementias have been less intensively researched and there are numerous inconsistencies in the results. In addition, the detected metabolic alterations are not disease specific. The heterogeneity of the studies conducted so far as well as methodological limitations lead to insufficient interpretation and comparability of the results. Large-scale, multi-center, cross-dementia MRS studies under standardized conditions and the use of new technologies are needed to overcome existing barriers in order to evaluate the potential benefits for dementia diagnosis and treatment.

## Author Contributions

All authors contributed to the article and approved the submitted version.

## Conflict of Interest

The authors declare that the research was conducted in the absence of any commercial or financial relationships that could be construed as a potential conflict of interest.
